# Recent updates for biomaterials used in total hip arthroplasty

**DOI:** 10.1186/s40824-018-0144-8

**Published:** 2018-12-05

**Authors:** Chang Yong Hu, Taek-Rim Yoon

**Affiliations:** Center for Joint Disease, Chonnam National University Hwasun Hospital, 160, Ilsim-Ri, Hwasun-Eup, Hwasun-Gun, Jeonnam 519-809 South Korea

**Keywords:** Hip, Arthroplasty, Biomaterials, Stainless steel, Cobalt-chromium alloy, Titanium alloy, Polyethylene, Ceramic

## Abstract

**Background:**

Total hip arthroplasty (THA) is probably one of the most successful surgical interventions performed in medicine. Through the revolution of hip arthroplasty by principles of low friction arthroplasty was introduced by Sir John Charnley in 1960s. Thereafter, new bearing materials, fixation methods, and new designs has been improved. The main concern regarding failure of THA has been the biological response to particulate polyethylene debris generated by conventional metal on polyethylene bearing surfaces leading to osteolysis and aseptic loosening of the prosthesis. To resolve these problems, the materials of the modern THA were developed since then.

**Methods:**

A literature search strategy was conducted using various search terms in PUBMED. The highest quality articles that met the inclusion criteria and best answered the topics of focus of this review were selected. Key search terms included ‘total hip arthroplasty’, ‘biomaterials’, ‘stainless steel’, ‘cobalt-chromium’, ‘titanium’, ‘polyethylene’, and ‘ceramic’.

**Results:**

The initial search retrieved 6921 articles. Thirty-two articles were selected and used in the review.

**Conclusion:**

This article introduces biomaterials used in THA and discusses various bearing materials in currentclinical use in THA as well as the newer biomaterials which may even further decrease wear and improve THA survivorship.

## Background

Total hip arthroplasty (THA) is one of the most popular surgical procedures performed worldwide. In England, the National Joint Registry recorded that more than 790,000 THAs were performed between 2003 and 2015 [1]. As of 2003, more than 200,000 THA operations were performed annually in the USA, about 2.5 million people are living with a hip replacement [2]. This number is expected to reach 572,000 by 2030 [3]. In Korea, the Health Insurance Review and Assessment Service informed that more than 60,000 THAs were performed between 2010 and 2017, and incidence was increasing over time [4].

Current developments in the field of artificial hip joints are focused on mechanical strength, biocompatibility [5–8], bioactivity [9–18] and materials that impart better wear resistance and mechanical reliability [19–28]. When an implant fails, patients may endure severe pain and disability and require revision surgery. Periprosthetic osteolysis is the primary cause of failure that is the result of activation of an innate immune response caused by wear of bearing materials in THA. Taken up by macrophages and multinucleated giant cells, the presence of wear debris particles may cause the release of cytokines, thereby resulting in inflammation that further activates osteoclasts and finally leading to implant loosening.

The functional goal of joint arthroplasty is to return a patient to activities of daily living and range of motion in the absence of pain. Thus, various biomaterials have been used and are constantly being developed. The purpose of this review was to provide an update on the development status of various materials in THA.

## History of development of Total hip arthroplasty

Metal on metal (MoM) bearings were made using large ball diameters during 1955–1965 [29]. However, the use of MoM bearings declined in the 1970s for some years after Sir John Charnley introduced a THA device based on metal on polyethylene (MoP) composed of a small metal ball and a cemented polyethylene (PE) cup in a 1960s [30]. Long term survival of these early implants was good, with around 77–81% of success rate 25 years after primary THA [31]. With the increasing use of THA in younger and more active patients, the revision rate becomes higher [32], and there has been concerns about the role of PE wear particles in osteolysis and loosening [31]. New materials have been introduced to prevent wear and osteolysis.

**Fig. 1:**
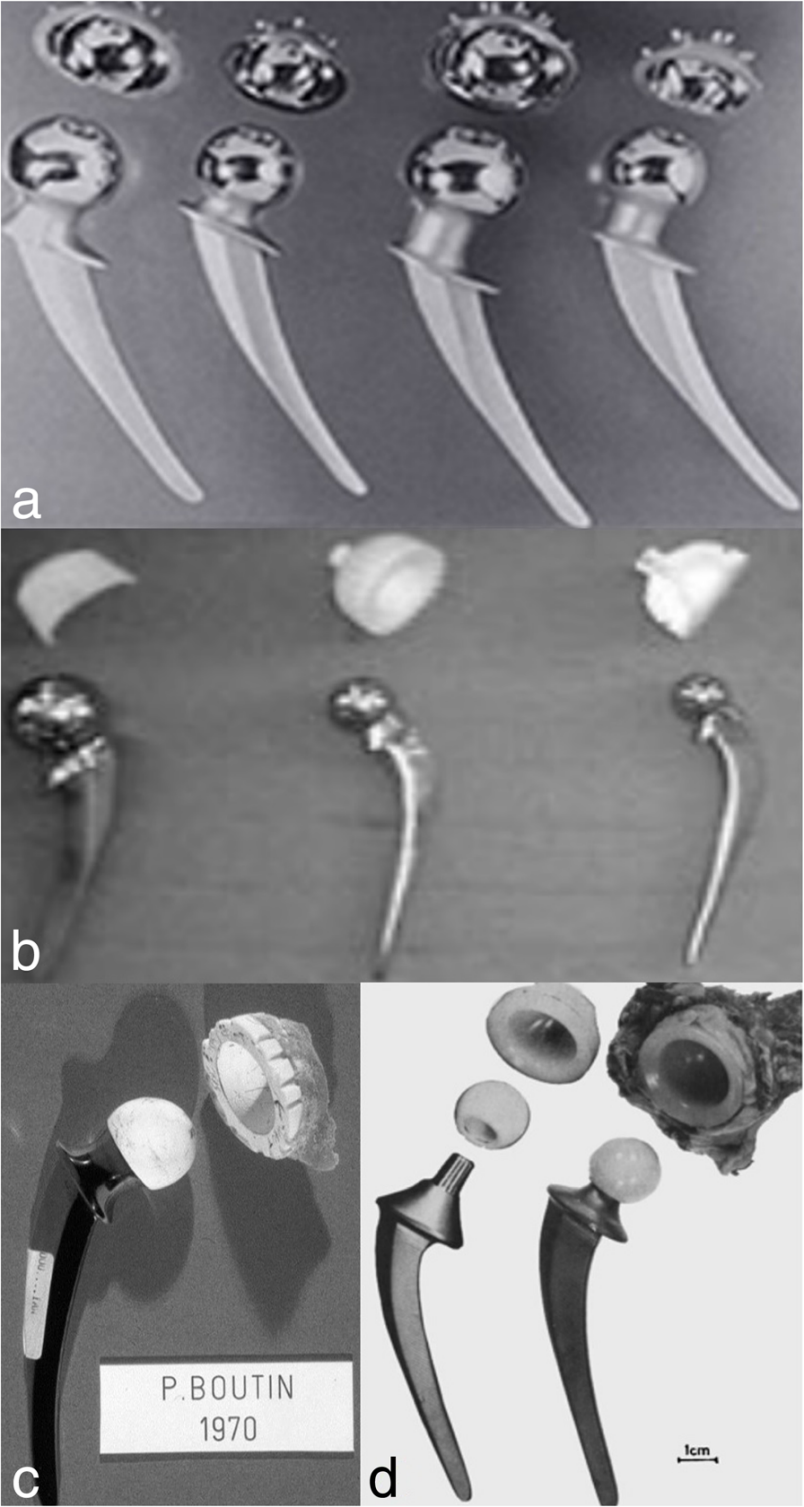
Early bearing materials used in THA (**a**) MoM Mckee-Farrer THA from 1960 (**b**) MoP combinations, Thompson prosthesis in a 1960s (**c**) CoC hip implants in a 1970s (**d**) CoP combinations over 1963–1973

Pierre Boutin, a French surgeon who anticipated the problem of “polyethylene disease”, began using alumina ceramic on ceramic (CoC) hip implants in a 1970s [33]. CoC implants have been used in THA and these developments also created ceramic on polyethylene (CoP) combinations as competitive bearing alternative along with MoM and CoC over 1963–1973 (Fig. 1).

Stainless steel was the first class of alloy introduced for orthopedic implants [34]. However, since some corrosion was inevitable, it has been recommended that stainless steel only be used for short duration purposes [35]. Currently, the most frequently used artificial hip joints are composed of an acetabular cup, liner, head and stem. The main materials for THAs were titanium, cobalt-chromium, PE, and ceramic, respectively.

## Supporting metallic materials

### Stainless steel

Stainless steels are iron-carbon based alloys. In general, these alloys contain Cr, Ni, Mo, Mn and C. The austenitic (316 series) alloys are typically used in fracture-fixation devices. The resistance to oxidation coupled with relative ease of machining, forming, and hardening makes stainless steel a strong candidate for material choice. Stainless steel is rarely used for THA material nowadays, because of poor biocompatibility, though stainless steel devices remain available in other countries (particularly the United Kingdom).

### Cobalt-chromium (co-Cr) alloys

Co-Cr alloys which was used in dentistry, are now one of the major materials used for hip prostheses. The favorable strength, corrosion, and wear characteristics make alloys of Co-Cr one of the main choice as an implant material. It is mainly used as cement type femoral stem material because the Young’s modulus is larger than titanium alloys and articulating head due to wear resistance.

### Titanium alloys

Titanium and its alloys are popular metallic implant biomaterials used in THA. Commercially, α + β titanium alloys, such as titanium-6Al-4 V have been the most commonly used alloys for stem and acetabular cementless components of THA, because of its comparatively low density, high mechanical strength, excellent corrosion resistance, and biocompatibility with bone [36].

However, Titanium alloys are not used for manufacturing of femoral head due to their poor wear resistance.

During the last two decades, vanadium free titanium alloys such as α + β titanium-6Al-7Nb alloy with improved biocompatibility have been developed by incorporating biocompatible elements such as Niobium [5–8]. Many researches have been devoted to the development of bulk metallic materials that have lower Young’s modulus, among which β titanium alloys have attracted significant attention.

### Alloy surface modifications

Classic implants are fabricated using traditional materials (sintered beads, fiber metal, plasma spray) which have several inherent biomaterial limitations. In order to achieve an effective osseointegration with a vital bone implant contact and reduce risk of loosening, the use of porous metals andcoatingswere developed [37]. In general, compared to stainless steels and Co-Cr alloys, titanium, some of its alloys and tantalum are the more suitable porous metallic materials used for orthopaedic applications.

Hydroxyapatite has been used in order to achieve the permanent mechanical fixation of an implant in the bone bed to involve the process of osseointegration [38]. Porous metal has been also introduced to obtain biological fixation and improve longevity of orthopedic implants [39]. The new generation of porous metal has intriguing characteristics that allows bone healing and high osteointegration of the metallic implants [40].

## Materials used in bearing surface

### Polyethylene

#### UltraHigh molecular weight polyethylene (UHMWPE)

UHMWPE was first introduced in 1962 as the bearing for the Charnley hip prosthesis. He developed the low-friction arthroplasty consisting of cemented fixation with a bearing surface of a 22.25-mm metallic femoral head andan all-PE cup [41].

Conventional PE is sterilized by gamma irradiation in air. This process offers the benefits of molecular crosslinking but can also produce free radicals that is oxidized in the presence of air [42]. Oxidation decreases resistance of the biomaterial, resulting in degradation and brittle PE, and thus may increase wear [43]. PE wear is multifactorial: among the different factors associated with wear are a patient’s higher activity level, a big femoral-head diameter or thin PE liners, vertical orientation of the acetabular cup, or the use of modular uncemented cups [44, 45]. UHMWPE wear debris mediated osteolysis is widely recognized as one of the most serious challenges in hip arthroplasty [46, 47].

#### High crosslinked UHMWPE (XLPE)

The developmentof new XLPE is aimed at improving UHMWPE in both cemented and uncemented implants. In order to decrease PE wear, research has attempted to improve wear resistance while maintaining mechanical properties and eliminating the oxidation process [48].

Crosslinking is accomplished by using either gamma radiation or electron beam radiation to break the molecular bonds. All manufacturers produce XLPE based on three processes: crosslinking, heat treatment, and sterilization while avoiding exposure to air. Higher crosslinking density is obtained using gamma irradiation or electron beams at a dose between 50 and 100 kGy to increase wear resistance. Heat treatment is aimed at eliminating free radicals that appear after crosslinking; this thermal treatment applies temperature above (remelting) or below (annealing) the melting transition temperature of the polymer (137 °C).

In vivo studies, Manning et al. reported 95% wear rate reduction, and Martell et al. showed 42% to 50% wear rate reduction using XLPE compared to conventional PE [49, 50]. Biologic activity of the wear debris was also reduced and osteolysis has been dramatically decreased [49–54].

#### Antioxidant doped polyethylene

In efforts to improve oxidation resistance without compromising mechanical properties through thermal treatments, XLPE is stabilized by the addition of antioxidants like vitamin E, to prevent oxidation of free radicals with the intention of increased wear resistance [19, 20, 55]. Although initial results are promising, longterm clinical results of this second generation PEs are not yet available.

#### Poly (2-methacryloyloxyethyl phosphorylcholine) (PMPC)

Kyomoto et al. made a great progress in tribological aspect of XLPE [21]. XLPE has been surface-treated on the articulating surface, covering the surface with a chemically thin layer (100–200 nm) to improve abrasion resistance. Poly (2-methacryloyloxyethyl phosphorylcholine) (PMPC), which is formed by photo-induced graft polymerization, creates a super-lubricious layer that mimicks articular cartilage [22]. A recent hip simulator study reported that MPC polymer grafted on the XLPE surface dramatically reduced the wear up to 70 million cycles [56].

### Ceramics

#### Alumina

Alumina has been used as a bearing surface in total hips since the 1970s [57]. Alumina ceramics have biocompatibility, high wear resistance, and chemical durability. Wear was as low as a few microns for a 15-year period in use, which is 2000 times less than a regular MoP sliding couple and 100 times less than a MoM prosthesis [58].

Although alumina ceramics have shown better wear characteristics than MoP, alumina has historically had a high incidence of fracture [59]. This high incidence of fracture led to improved manufacturing processes which was possible by decreasing grainsize and porosity, and by tempering process for the increase of toughness [60].

With the improvements made in alumina material properties, the incidence of fracture has declined dramatically in recent years. The decreased incidence of fracturing of alumina components has made ceramics a more feasible option, especially for younger, more active patients [59].

#### Zirconia

Zirconia femoral heads were introduced in Europe in 1985 and later introduced into the USA in 1989 [61]. The move from alumina to zirconia as a femoral head component was because of the high incidence of fractures of alumina heads and the increased fracture toughness of zirconia compared to alumina [62]. Zirconia also had a historically higher bending strength than alumina [63].

However, in view of the recently reported potential for zirconia ceramics to undergo monoclinic phase transformation in vivo, with resultant increased fracture risk and degradation of wear properties [64, 65]. Unfortunately, the largest manufacturer of zirconia femoral heads recalled their products in 2001, because of problems with the thermal processing associated with those batches [61]. Since the recall, use of zirconia stabilized with yttria has declined, but a trend toward developing alumina-zirconia composites to improve performance of ceramic bearings has emerged [66].

#### Alumina-zirconia composites

Despite the long clinical history of alumina and zirconia in THA, both materials had drawbacks. Attempts to overcome the weaknesses of these materials by combining alumina’s hardness with zirconia’s toughness have led to the development of zirconia-toughened alumina (ZTA), which was first commercialized by CeramTec under the trade name of BIOLOX® Delta in around 2000. ZTA is an alumina matrix composite containing 75% fine grained alumina of 0.5–0.6 μm in diameter and 25% Y-TZP with a grain size of 1 μm or smaller to obtain a flexural strength of 1200 MPa and a fracture toughness of 6.5 MPa√m [66]. The base alumina matrix ensures high hardness of the materials, and the addition of zirconia particles promotes resistance to crack propagation [62]. ZTA also slows down the kinetics of hydrothermal aging, which is a potential advantage over monolithic zirconia.

#### Silicon nitride

Silicon nitride is a non-oxide ceramic material with high strength and toughness and has been used as bearings, turbine blades for more than 50 years. In the medical field, since 2008, it has been used in cervical spacer and spinal fusion devices, with few adverse reports among 25,000 implanted spinal cages [67, 68]. Silicon Nitride has been recently regarded as a bearing material for artificial hips due to its high biocompatibility, moderate Vickers hardness of 12–13 GPa, Young’s modulus of 300 GPa, high fracture toughness of 10–12 MPa√m and flexural strength of 1 GPa, with a typical grain size of 0.6 μm after alloying with small amounts of yttria and alumina [69]. Mechanical testing has shown higher fracturetoughness, higher flexural strength, higher resistance to hydrothermal degradation. Biocompatibility tests haveshown that Si3N4 does not produce any adverse reactions behaving similar to alumina [70].

Recent hip simulator studies show that self-mated silicon nitride couples exhibit up to 3 million cycles of wear compared to self-mated alumina; however, some self-mated silicon nitride couples show increased wear at the end of 5 million cycles compared to alumina CoC [71]. Further long term clinical studies of retrieved heads of silicon nitride and hip simulator studies by others may be necessary.

### Hybrid Design of Oxide Ceramic Layer on metal (Oxinium™)

A new zirconium alloy (Zr-2.5Nb) was introduced to hip arthroplasty in 2003 [68]. When heated in an air environment, the surface of the metal zirconium converts to a black zirconium oxide which is approximately 4 to 5 μm thick [60, 72, 73]. This oxidized zirconium femoral head commercialized as Oxinium™ (OxZr; Smith & Nephew, Memphis, TN, USA) is not a coating, but a surface transformation by oxygen diffusion hardening process, which is expected to provide improved resistance under load bearing. It is a relatively new material used as an alternative to alumina or zirconia ceramics, demonstrating increased hardness and decreased surface roughness similar to zirconia, but possessing inherently high fracture toughness and fatigue strength because of the metal substrate [74].

In a simulator study, it was observed that Oxinium™ heads produced 45% less wear than did smooth Co-Cr heads, and, when the heads were roughened, the difference was much greater, with oxinium producing 61% less wear. Lewis et al. compared 50 Co-Cr and 50 oxinium heads and observed the clinical outcome to be equivalent at 2 years of follow-up [75].

Despite the clinical use of OxZr’s head for more than eight years, we need more reliable data about long term outcomes.

### Ultra-hard coatings on metals

While Co-Cr alloy in self-mated configuration or the alloy heads sliding against PE or XLPE are frequently used in THA, over 50% of failed artificial hipjoints are mainly due to osteolysis mediated aseptic loosening in addition to metal ion allergies overa long term period [76]. A frequent used alternative hybrid approach is to coat metal alloys with very hard, biocompatible surface layers such as diamond-like carbon (DLC, 5000 HV) [77] or titanium nitride (TiN 2100 HV) [78].

This approach ensures that the original properties of high strength metallic substrate are retained while: (a) supporting a bearing surface; and (b) avoiding the release of toxic metal ions from the underlying the Ti alloy substrate. However, there are several problems such as local delamination, crevice corrosion, third body wear [78, 79]. Another method is to deposit pure diamond on the metal head. In this regard, coating of ultra nanocrystalline diamond (UND) with grain size of 3–100 nm was directly applied to Ti and Co-Cr alloy using microwave plasma CVD [80, 81]. UND coatings possess high hardness (56–80 GPa) and low surface roughness, high wear resistance to third-body wear particles [82]. Nevertheless, large compressive stresses are retained in the UND coating due to impurities at the grain boundaries, affecting the adhesion to the substrate [83]. In short, further enhancements to these coating techniques are needed to meet the high wear resistance, mechanical reliability and adhesive requirements for prolonged THA.

## Clinical aspects of bearing surface

**Fig. 2:**
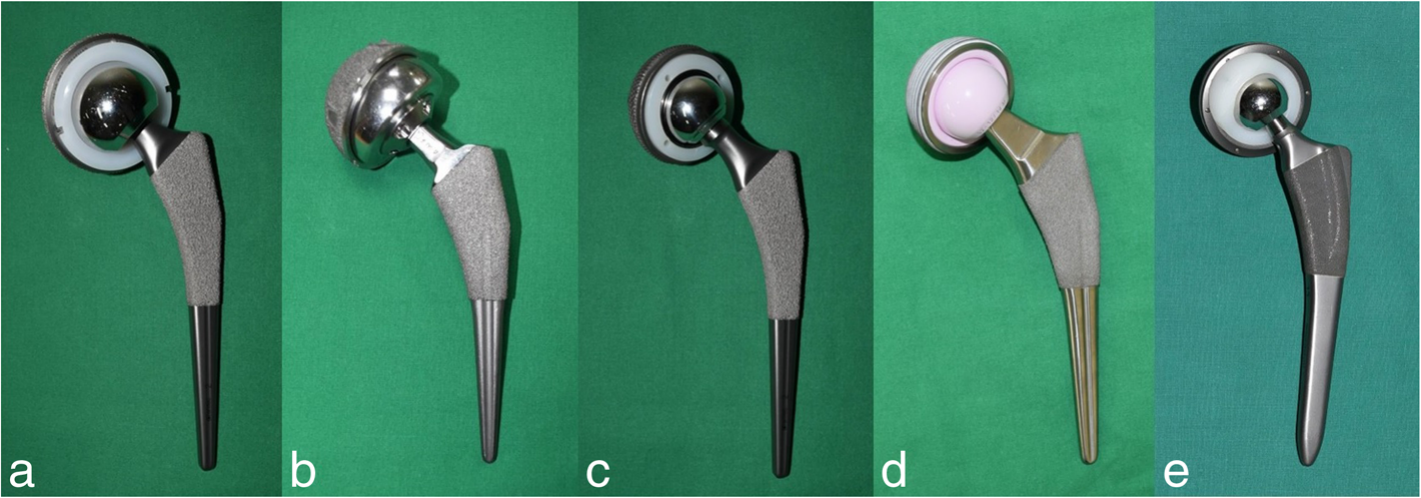
Recent bearing materials used in THA (**a**) MoP bearing (**b**) Large head MoM bearing (**c**) Small head MoM bearing (**d**) CoC articulation (**e**) CoP articulation

Bearing couples should have a low coefficient of friction, high surface hardness with lowductility and scratch resistance, and generate a low volume of wear particles. Moreover, surfaces exposed to tissues should be non-cytotoxic, biocompatible, and bioinert [84]. There are several bearing materials that are commonly used in clinical practice (Fig. 2).

### MoP articulation

#### Advantages

MoP composed of a small metal ball and a cemented PE cup in 1963 [85]. Over the last few decades, one of the most acceptable bearing surface couple in a prosthetic hip is a Co-Cr femoral head articulating with a UHMWPE acetabular component in view of the excellent Long term results available. Tsukamoto M et al. reported that XLPE group presented a significantly reduced wear rate compared with the conventional PE group (XLPE groups, 0.035 mm/yr.; conventional PE group, 0.118 mm/yr) [86]. This bearing surface couple remains the one of the standards to which wear testing for other bearing articulations are compared. MoP bearing surface, a bearing surface with good long term results in elderly patients, once was taken as gold standard for THA [87].

#### Disadvantages

**Fig. 3:**
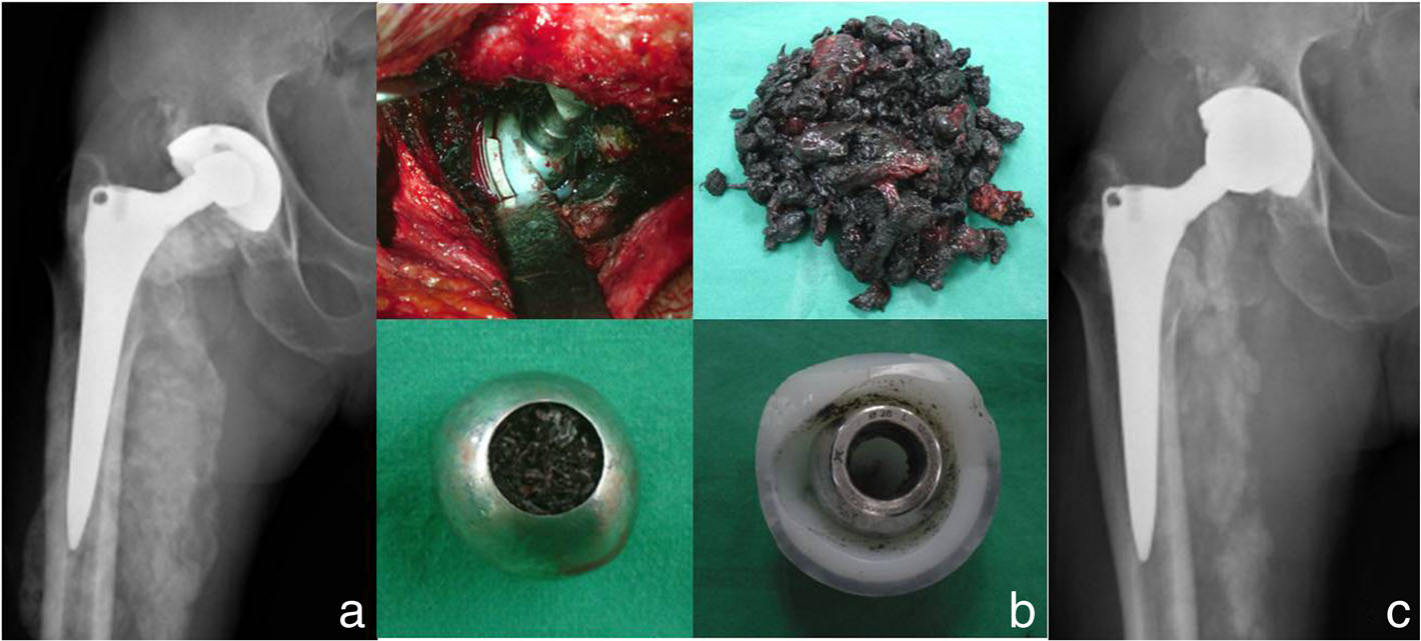
A 62-year-old male patient with right total hip arthroplasty using MoP bearing (**a**) Radiograph illustrating liner wear and metalosis (**b**) Severe metalosis and osteolysis (**c**) Radiographs after revision surgery including excising mass, changing to metasul liner and metal head after cementing

It became clear that PE liner wear debris generated with time was associated with the occurrence of osteolysis which leads to subsequent loosening and eventual implant failure (Fig. 3). This osteolysis appears tooccur more commonly at wear rates of more than 0.1 mm/yr. and is uncommon when wear rate is less than 0.05 mm/yr. [88, 89]. It has been reported that the osteolysis rate of MoP is as high as 26%, and aseptic loosening rate is 3% at 10-year follow up [90].

During the past decade, different manufacturers have begun to develop new biomaterials in order to decrease PE wear, such as XLPE, Antioxidant Doped Polyethylene and PMPC. Brach et al. reported better performance by this newer XLPE than with conventional or even first-generation XLPE [91]. The other strategy is to introduce vitamin E, the antioxidant alpha-tocopherol, into UHMWPE prior toconsolidation to help prevent the oxidative degradative reaction. This would avoid the deleterious effect of the melting process that decreases the mechanical properties of PE. Oral et al. reported good wear and improved mechanical and fatigue properties [92]. However, these new technology whose success and impact will be determined in the longer term. Analysis of retrieved components and clinical results will continue to inform us on the effects of wear problems [93].

#### Wear mechanism

Adhesive features have been found on the surface of PE cups matched with a metallic ball [94]. Welding between the cup and ball generates fibrils on the surface of the polymeric material. These fibrils may become torn off and pulled away as loose particles. Without sufficient lubrication, bigger fragments may be transferred from counterbody to body and vice versa. Such particles may introduce abrasion in the form of two or three body abrasion resulting in scratches on the surface.

### MoM articulation

#### Advantages

Proposed advantages included the reduction in wear, improved range of movement and a lower dislocation rate [95, 96] and MoM bearings have wear rates that are 20 to 100 times lower than metal-on-conventional polyethylene [97]. MoM THA using a 28 mm head has shown favorable results compared with large head MoM THA. Small head MoM showed a relatively low rate of aseptic loosening at a mean follow up of 20 years [98]. Yoon et al. reported that good clinical results with no complicationsin THAs with MoM bearing even with chronic renal failure [99]. Small head MoM bearing seems to have good results, relatively.

#### Disadvantages

The problems with large bead MoM began to appear in 2005. With increasing clinical experience, the national joint registries have recently reported the failure rate of THA with MoM bearings to be 2–3 fold higher than contemporary THA with non MoM bearings [100, 101] associated with local bone and softtissue necrosis, with pseudotumor formation comprising a predominantly lymphocytic inflammatory reaction [102, 103] and, wear particles in the form of cobalt and chromium ions have been detected throughout the body [104]. Although granuloma have been found in both the liver and spleen [105] and increased chromosomal translocation has been found within lymphocytes [106], there is currently no hard evidence that this leads to neoplasia [107].

Furthermore, midterm studies demonstrated increased rates of osteolysis and implant.

**Fig. 4:**
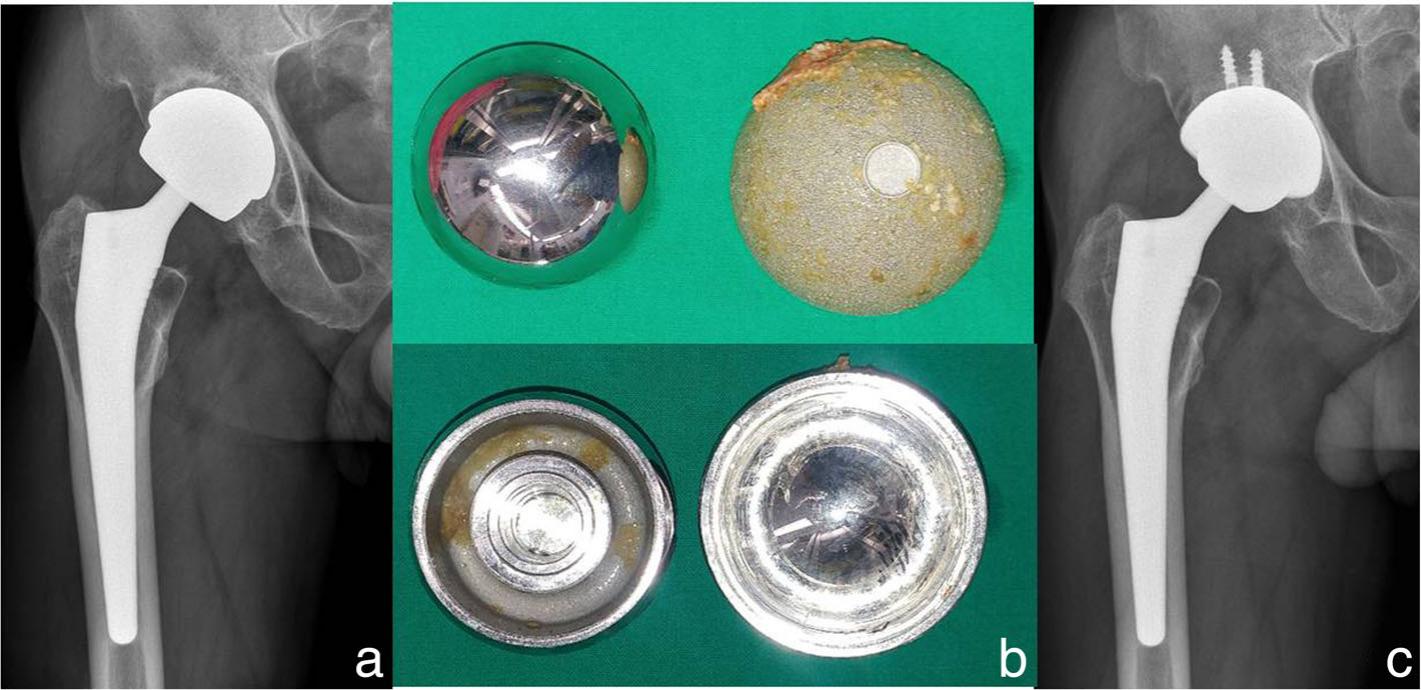
A 68-year-old male patient with right total hip arthroplasty using large head MoM bearing (**a**) Preoperative radiograph of acetabular aseptic loosening (**b**) Large head MoM bearing (**c**) Radiographs after acetabular revision using CoC bearing

Failure (Fig. 4), raising concerns about the longevity and safety of this bearing surface [108–110]. Korovessis et al. followed 217 patients who underwent a primary THA using a second-generation, large diameter MoM bearing surface for an average of 77 months [108]. During this follow up period, 14 THAs (6.5%) were revised and found to have concerning signs of metallosis and lymphocytic infiltrates raising concerns about this bearing surface. Park et al. followed 169 hips who underwent THA using a second-generation MoM bearing surface for a minimum of 24 months and noted 10 hips (5.9%) had early osteolysis [110]. The poor performance associated with large head MoM bearing surfaces led the Food and Drug Administration to remove several second-generation MoM THA systems from the market, effectively ushering out the era of this bearing surface [111].

#### Wear mechanism

The dominant wear mechanism is determined to be mild surface fatigue. Surface fatigue is introduced by direct solid contact of surface asperities or by foreign and/or system inherent third bodies, which repeatedly slide or roll within the wear track. Although these third bodies contribute to fatigue related wear loss, this wear is several orders of magnitude smaller than would be introduced by adhesion. Tribochemical reactions also comprise an important wear mechanism in MoM hip joints. They might be triggered by the synergistic interaction of wear and corrosion and can influence the tribosystem in a positive or negative manner.

### CoC articulation

#### Advantages

In the late 60s, CoC bearings were first introducedin hip arthroplasty by Boutin [112]. They have undergone many generations of changes since then during which the susceptibility to fracture (a problem in early generation ceramics) has been overcome. Since ceramics are harder than metals, are biologically inert and have better lubrication properties leading to low wear rates [113], CoC bearings make an attractive choice for ensuring long term survival of hip prosthesis. The minimal wear particles released from CoC bearings are also biologically relatively inert and at nanometric size, significantly reducing the osteolysis produced due to PE wear particles. In addition, CoC bearing combination also has lesser coefficient of friction, higher wettability with biologically inert wear particles [114]. Clinical results have confirmed higher survivorship, lesser wear and low osteolysis making these bearings an excellent choice for young and active individuals [115]. Yoon et al. reported no case of osteolysis after 3^rd^generation of CoC bearing THA [116] and lower rate of osteolysis has been confirmed by many other studies [117, 118].

Hernigou et al. investigated wear and osteolysis in bilateral arthroplasties (one CoC and the contralateral CoP) of patients who had survived 20 years without revision and without loosening of either hip [119]. The number of lesions was higher on the side with Cop couple. Hai-bo Si et al. reviewed several articles that wear rate was also lower in CoC than CoP THA [120].

CoP articulations also reportedly have reduced wear rates compared to metal heads on PE in THA [121].

#### Disadvantages

**Fig. 5:**
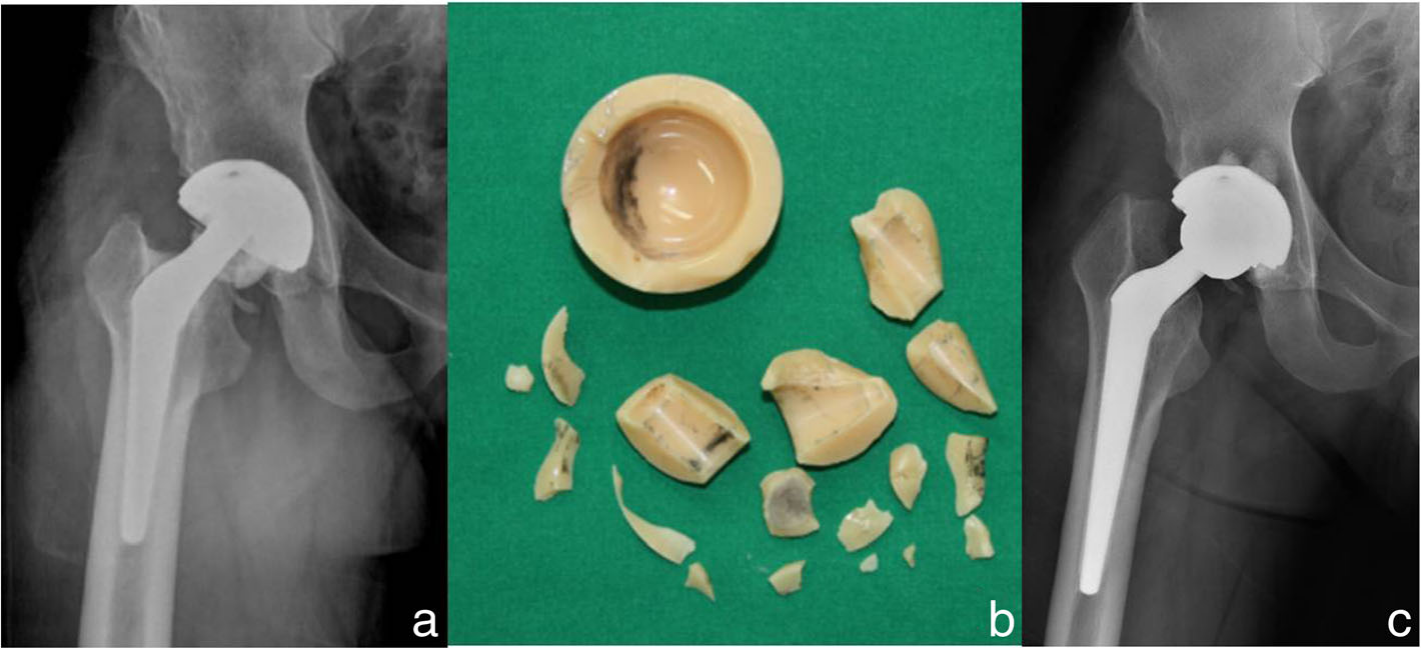
A 34-year-old male patient with right total hip arthroplasty using CoC articulation (Forte) (**a**) Radiograph with fractured ceramic head and liner (**b**) The fractured ceramic head and liner (**c**) Radiographs after revision surgery changing the ceramic liner and fractured head to metasul liner and metal head after cementing

Though the ceramics are the new preferred bearing surface, especially in the young, they are not without their share of complications which include squeaking noises, stripe wear, a rare bearing surface fracture or chipping during insertion. Complications have been more commonly associated with acetabular component malposition (more vertical cups), smaller femoral heads and non-adherence to meticulous surgical technique [122, 123]. Fracture of a ceramic head and/or liner remains a major disadvantage for this bearing combination compared with MoP or MoM (Fig. 5). Earlier generations of alumina ceramic heads had a reported risk for fracture until 13.4%, however for newer implants (Biolox Forte and Delta) the reported fracture rate is much lower at 0 to 3.2% [124, 125].

Another concern remains squeaking of ceramic bearings. This potentially affects the patient’s quality of lifeand survivorship of the implant due to revision of the squeaky hip. Noises emanating from ceramic bearings (usually clicking and squeaking) have been reported with rates that vary from 0 to 33%. Fortunately clinically the problem is often minor in themajority of patients and revision surgery is indicated onlyoccasionally. Yoon et al. also reported low incidence of squeaking (1.5%), and there were no complications to limit daily life and no revision [126]. Despite these shortcomings, CoC articulation seems to be the best recently.

#### Wear mechanism

The dominating wear mechanism is mild surface fatigue maintaining a polished appearance in most areas of the articulating surfaces. The grain structure of the material can be easily identified in such polished areas. Sometimes, fine scratches originating from the initial polishing procedure during manufacturing are still visible indicating a very mild wear process. Abrasive scratches can be observed, however to a much lower extent than in other systems. No tribochemical reaction layers have been reported.

### Ceramic on PE (CoP) articulation

#### Advantages

CoP as a bearing couple currently accounts for around one in seven hip replacements in the UK [127]. Potentially this keeps the advantages of the softer, less rigid PE surface and utilises the advantages of the smooth, hard ceramic surface.

Over the period examined, CoP bearing surfaces steadily increased in popularity to become the most popular bearing surface type. Although concerns about fracturing of the femoral head [128] and increased costs had decreased usage of ceramic heads in the 1980s and 1990s, the advent of large ceramic heads with low fracture rates, low wear rates, and multiple neck length options over the past decade had increased the use of CoP bearings [129].

It is also apparent from the literature that CoC hips have lower wear rates compared with CoP hips, however, the mid-term studies utilising newer alumina ceramic with newer PEs show no difference in osteolysis or patient satisfaction at five years [130].

#### Disadvantages

Theoretically, the limitations of CoP bearing surfaces involves the risk of alumina head fracture, the resultant difficult revision surgery [131], metal transfer which can increase surface roughness, and third body wear leading to increased PE wear [132]. With the advent of delta ceramic, the rate of fracture decreased dramatically. There has been no reports yet, about the clinically significant problem coming from metal transfer (Table 1).

**Table 1:** Advantages and disadvantages of bearing surfaces

Bearing Surface	Advantages	Disadvantages
MoP Articulation	∙ Good long term results in elderly patients	∙ Higher rate of liner wear
	∙ Newly materials - XLPE, Antioxidant doped PE	∙ PE liner wear debris generated the occurrence of osteolysis
		∙ Newly materials do not have long term results
MoM Articulation	∙ Reduction in wear	∙ Bone and soft tissue necrosis with pseudotumor formation
	∙ Improvement of range of movement	∙ Cobalt and chromium ions can affect the body
	∙ Lower dislocation rate	∙ Relatively high rate of osteolysis and implant failure
	∙ Good clinical results in small head MoM	∙ Withdrawal of large head MoM
CoC Articulation	∙ Lower wear rate	∙ Ceramic fracture
	∙ Lower osteolysis	∙ Squeaking noise
	∙ Very higher survivor rate in long term results	
	∙ Harmless wear particle to human body	
CoP Articulation	∙ Ceramic surfaces advantages + PE surfaces advantages	∙ Alumina head fracture
	∙ Lower wear rate	∙ Metal transfer

#### Wear mechanism

It may be similar to MoP articulation. Wear mechanism is surface fatigue where the PE part is usually by far more affected than the hard counterbody. Surface fatigue is associated with repetitive loading and generates wear features such as pitting and delamination [133, 134]. The most common wear appearance in PE cups is polishing.

Unlike in MoM articulation, no tribochemical reactions have yet been reported for polymer cups. But, this does not preclude their existence. PE transfer films on the hard counter parts have been reported [135].

### Orthopedic wear debris

Wear debris is formed at prosthetic joint articulations, at modular interfaces, at areas of impingement, and at nonarticulating interfaces due to abrasion with the surrounding bone, or debris [136].

Cells in the periprosthetic environment are exposed to a continuous production of wear particles. The biologic response to particle wear debris complex and drives the process toward periprosthetic tissue destruction and implant loosening. Although most of the studies have focused on UHMWPE particles, particles generated from other sources may induce an inflammatory reaction and subsequent osteolysis [137, 138]. For example, silicate and stainless steel particles, as possible containments from drilling and reaming tools, may elicit an aggressive cellular response. Although they may participate in initiating and/or instigating an inflammatory process, their role is considered minor. Alumina ceramic is a material commonly described as bio-inert [139]. However, submicron-sized particulates of alumina and zirconia may elicit a similar but less intense reaction to those seen with submicron-sized polymers and metal debris.

## Conclusion

THA remains a highly successful procedure providing good pain relief and improvement of activity levels. Despiteits success, the expectations continue to increase with more and more young patients undergoing hip replacement and most of them seeking higher activity level (higher range ofmotion and stability in those ranges) as well as longevity of the prosthesis. Besides, the fixation method for the prosthesis, good surgical approach, bearing surfaces remain the most important determinant of longevity of the hip prosthesis.

Newer bearing surfaces incurrent clinical practice have shown promising clinical outcomes. With success of these wear reducing bearing surfaces, the scientific community will need to focus on not only further reducing abrasive wear but on reducing stress shielding as well by newer materials as well as designs. Ongoing research and the future of biomaterials in the hip are anticipated.

## Abbreviations

CoC: ceramic on ceramic
Co-Cr: cobalt-chromium
CoP: ceramic on polyethylene
DLC: diamond-like carbon
MoM: metal on metal
MoP: metal on polyethylene
PE: polyethylene
PMPC: poly (2-methacryloyloxyethyl phosphorylcholine)
THA: total hip arthroplasty
TiN: titanium nitride
UHMWPE: ultra high molecular weight polyethylene
UND: ultra nanocrystalline diamond
XLPE: high crosslinked UHMWPE
ZTA: zirconia-toughened alumina
